# Veterinary students’ perceptions of their learning environment as measured by the Dundee Ready Education Environment Measure

**DOI:** 10.1186/1756-0500-7-170

**Published:** 2014-03-24

**Authors:** Jacquelyn M Pelzer, Jennifer L Hodgson, Stephen R Werre

**Affiliations:** 1 Office of Academic Affairs, Virginia Maryland Regional College of Veterinary Medicine, Phase III 245 Duckpond Drive, Virginia Tech, Blacksburg, VA 24601, USA; 2 Office of Research and Graduate Studies, Virginia Maryland Regional College of Veterinary Medicine, Phase III 245 Duckpond Drive, Virginia Tech, Blacksburg, VA 24601, USA

**Keywords:** Learning environment, Veterinary students’ perceptions, DREEM, Reliability, Validity

## Abstract

**Background:**

The Dundee Ready Education Environment Measure (DREEM) has been widely used to evaluate the learning environment within health sciences education, however, this tool has not been applied in veterinary medical education. The aim of this study was to evaluate the reliability and validity of the DREEM tool in a veterinary medical program and to determine veterinary students’ perceptions of their learning environment.

**Methods:**

The DREEM is a survey tool which quantitatively measures students’ perceptions of their learning environment. The survey consists of 50 items, each scored 0–4 on a Likert Scale. The 50 items are subsequently analysed within five subscales related to students’ perceptions of learning, faculty (teachers), academic atmosphere, and self-perceptions (academic and social). An overall score is obtained by summing the mean score for each subscale, with an overall possible score of 200.

All students in the program were asked to complete the DREEM. Means and standard deviations were calculated for the 50 items, the five subscale scores and the overall score. Cronbach’s alpha was determined for the five subscales and overall score to evaluate reliability. Confirmatory factor analysis was used to evaluate construct validity.

**Results:**

224 responses (53%) were received. The Cronbach’s alpha for the overall score was 0.93 and for the five subscales were; perceptions of learning 0.85, perceptions of faculty 0.79, perceptions of atmosphere 0.81, academic self-perceptions 0.68, and social self-perceptions 0.72. Construct validity was determined to be acceptable (p < 0.001) and all items contributed to the overall validity of the DREEM. The overall DREEM score was 128.9/200, which is a positive result based on the developers’ descriptors and comparable to other health science education programs. Four individual items of concern were identified by students.

**Conclusions:**

In this setting the DREEM was a reliable and valid tool to measure veterinary students’ perceptions of their learning environment. The four items identified as concerning originated from four of the five subscales, but all related to workload. Negative perceptions regarding workload is a common concern of students in health education programs. If not addressed, this perception may have an unfavourable impact on veterinary students’ learning environment.

## Background

Veterinary medical education in the United States is currently facing a paradigm shift with calls for greater emphasis on competency-based education. These competencies include a range of professional or non-technical attributes to address heightened expectations of the veterinary profession [1-3]. This broadening of veterinary curricula is in the face of rapidly expanding knowledge across all animal species for which veterinarians are responsible. As a result, many veterinary curricula have become increasingly demanding, with associated increases in workload and intensity often culminating in elevated stress and lack of motivation in veterinary students [4,5]. However, more in-depth appraisal of students’ perceptions of the current learning environment have not been performed in veterinary programs to identify specific areas of concern. In health sciences education, the environment in which students learn has been shown to influence a number of educational outcomes including academic achievement, course satisfaction, student aspirations, as well as sense of well-being [6-11]. In these settings the Dundee Ready Education Environment Measure (DREEM) is the most common tool used to evaluate students’ perceptions of their learning environment [12]. For example, this tool has been used to assess the impact of curricular change and interventions [13-15], as well as identifying strengths and weakness of curricula with a view to introducing change [16,17].

The DREEM is a 50-item questionnaire which measures students’ overall perceptions of their learning environment as well as five different focus areas or subscales; students’ perceptions of learning, students’ perceptions of course organizers (faculty), students’ academic self-perceptions, students’ perceptions of atmosphere, and students’ social self-perceptions. This tool has been translated into at least ten languages [18] and applied in multiple healthcare professional settings in over twenty countries [19]. The different healthcare professional programs where this instrument has been utilized includes medical (undergraduate and postgraduate), dental, nursing, and chiropractic [20-24], but no veterinary programs have been investigated to date. This is despite the fact that veterinary medical programs have many similarities in their structure and degree of difficulty to other healthcare programs, especially human medicine. However, when using an evaluation instrument in a different context it is important to determine its reliability and validity in the new environment. Furthermore, inconsistent reporting of statistical analyses, and variable results in other studies, has led to calls for further psychometric evaluation of the DREEM instrument [12,19].

We were also interested in applying this instrument in our educational setting as a number of factors inherent to United States veterinary educational systems could influence students’ perceptions of their learning environment and influence DREEM results. For example, veterinary education is predominantly post-graduate with the majority of students having completed at least 60 credit hours of undergraduate coursework. This is in contrast to undergraduate medical programs outside the United States in which students may enter immediately following high school. In addition, the gender ratio in veterinary programs is approximately 80% female, while in medical schools this ratio is usually lower. Students’ perceptions also may be influenced by age, where the average age of students entering our program is 24, considerably older than students in undergraduate medical programs. Furthermore, many of our students are pursuing veterinary medicine as a second career, again in contrast to undergraduate medical education programs. For these reasons we wished to evaluate the DREEM in a veterinary medical program in the United States.

The aims of this study were; 1) to evaluate the psychometric properties of the DREEM in the context of veterinary medical education, and 2) to determine students’ perceptions of their learning environment within a United States veterinary program and identify potential areas of student concern.

## Methods

### Doctor of Veterinary Medicine (DVM) program

The Virginia Maryland Regional College of Veterinary Medicine (VMRCVM) is an accredited veterinary program within the United States. The College offers a four year, Doctor of Veterinary Medicine (DVM) degree with most students starting after completion of a four year undergraduate degree. The program comprises three years (six semesters) of pre-clinical training and a final year of work place-based training in a variety of clinical settings including a veterinary teaching hospital. The VMRCVM has a traditional curriculum (non-problem based learning) with additional courses being offered to allow students to focus in areas of interest (tracks) above and beyond the core curriculum. However, as there is no limited licensure within veterinary practice, students must demonstrate competencies within each major animal species area. There are a total of 419 students within the DVM program, approximately 80% being female, with an average age of 24 upon entry.

### Participants and data collection

In January 2013, all students (n = 419) enrolled in the DVM program were invited to participate in a self-administered, on-line DREEM survey. The survey remained available for one week. An email to the students was used to convey background information on the DREEM, instructions for completing the survey, and how results would be used. Anonymity of the survey was emphasized. Prior to their participation, students were invited to attend an optional twenty minute presentation to provide further explanation of the DREEM tool and its purpose. Attendance was not monitored at this presentation. The study was approved by the Office of Research Compliance at Virginia Tech (IRB#12-957).

### Dundee ready education environment measure

The DREEM is a survey tool developed to quantitatively measure student’s perceptions of their learning environment within a health profession educational setting [18]. The DREEM survey consists of 50 items, or statements, each scored 0–4 on a 5-point Likert Scale (0 = strongly disagree to 4 = strongly agree) [25]. There are nine negatively stated items (questions 4, 8, 9, 17, 25, 35, 39, 48 and 50) which are scored in reverse (0 = strongly agree to 4 = strongly disagree). Mean scores for individual items are calculated with a maximum score of 4.0 for each item. The 50 items are subsequently analysed within five subscales created by combining specific items [25]; 1) students’ perceptions of learning (12 items, maximum score 48), 2) students’ perceptions of faculty (11 items, maximum score 44), 3) students’ academic self-perceptions (8 items, maximum score 32), 4) students’ perceptions of atmosphere (12 items, maximum score 48) and 5) students’ social self-perceptions (7 items, maximum score 28). An overall or composite score is obtained by summing the mean score for each subscale, with a maximal possible score of 200.

An approximate guide to interpretation of the mean overall score, the subscale scores, and scores for individual items has been established by the developers of the DREEM (Table 1) [26].

**Table 1:** **Interpretation of mean scores as defined by the DREEM developers**[26]

**Mean Score**	**Score**	**Interpretation**
Total mean score	0-50	Very poor
	51-100	Significant problem
	101-150	More positive than negative
	151-200	Excellent
Subscale mean score		
*Students’ perceptions of learning*	0-12	Very poor
	13-25	Negatively viewed teaching
	25-37	A more positive perception
	37-49	Teaching highly regarded
*Students’ perceptions of faculty (teachers)*	0-11	Very poor
	12-22	Needs re-education
	23-33	Moving in the right direction
	34-44	Model instructors
*Students’ academic self-perception*	0-8	Feelings of total failure
	9-16	Many negative aspects
	17-24	Feeling more on the positive side
	25-32	Confident
*Students’ perceptions of atmosphere*	0-12	Very poor environment
	13-24	Many issues need changing
	25-36	A more positive attitude
	37-48	A good overall feeling
*Students’ social self-perception*	0-7	Miserable
	8-14	Not a nice place
	15-21	Not too bad
	22-28	Very good socially
Individual items mean score	≤2	Problem areas
	2.1-3	Needs improvement
	3.1-3.5	Positive aspect
	>3.5	Excellent

In this study, the DREEM survey was used in its original language with the exception of two terms which were not familiar to veterinary students in the United States. “Course organisers” was substituted with “faculty”, and “registrar” was replaced by “student”.

### Statistical analysis

Normal probability plots showed that scores for each of the items, composite scores of the five subscales, and the overall composite score followed an approximately normal distribution. For the individual items, scores increased in a step-plot pattern without showing minimum or maximum inflation. For the composite scores, near straight lines were observed. Subsequently, means and standard deviations were calculated for all 50 items as well as the five subscales and the overall score for the entire student sample.

The parametric measure of internal consistency (Cronbach’s alpha) was determined for the overall score and each subscale using PROC CORR. The following guide for interpretation of Cronbach’s alpha was used; α ≥ .90 = excellent, 0.70-0.89 = good, and 0.60-0.69 = acceptable [27]. An acceptable or higher Cronbach’s alpha demonstrates that the individual items that constitute a test, the DREEM tool in this case, correlate well with one another or with the test total.

To examine construct validity of the DREEM in this setting a confirmatory factor analytic model was fitted to the data using PROC CALIS. This analysis was used to measure whether the construct was consistent with a hypothesized measurement model. In our model we specified the five subscales as latent variables (structural variables) and the 50 items as manifest variables (indicator variables). Model parameters were estimated using maximum likelihood. Four criteria [28] were inspected to assess model fit; the ratio of goodness of fit chi-square to the degrees of freedom, the Bentler Comparative Fit Index, the Bentler-Bonett Non-normed Index, and inspection of the standard errors for the loading factors to verify that none had a value near 0.

Finally, standardized and unstandardized *t* values were examined to assess whether the indicator variables were measuring the underlying constructs of interest (i.e., perceptions of learning, perceptions of faculty, academic self-perceptions, perceptions of atmosphere, and social self-perceptions). All analyses were performed using SAS version 9.3 (Cary, NC, USA).

## Results

There were 224 respondents who fully completed the survey (53% response rate), with 179 females and 45 males which reflect the gender distribution of our student population.

Statistical analysis of the collected data determined the internal consistency (Cronbach’s α) for the overall score to be 0.93, and for the five subscales; 1) students’ perceptions of learning = 0.85, 2) students’ perceptions of faculty = 0.79, 3) students’ academic self-perceptions = 0.68, 4) students’ perceptions of atmosphere = 0.81, and 5) students’ social self-perceptions = 0.72 (Table 2). Results for confirmatory factor analysis of the construct validity are recorded in Table 3. Based on the model fit criteria evaluated, we concluded the model fit was adequate. Unstandardized and standardized *t* values for all 50 items were greater than 3.291 and therefore significant at p < 0.001 [28]. All items contributed to the overall validity of the DREEM.

**Table 2:** Results for the overall DREEM score and the five subscale scores

**Veterinary students (n = 264)**	**Sum of mean scores**	**Maximum possible score**	**Interpretation of sum of mean scores using developers’ descriptors**[26]	**Cronbach’s α (Internal consistency)**
DREEM overall score (sum of subscale means)	128.9	200	More positive than negative	0.93
Students’ Perceptions of Learning (sum of item means: 1, 7, 13, 16, 20, 21, 24, 25, 38, 44, 47 and 48)	30.6	48	More positive perception	0.85
Students’ Perceptions of Faculty (sum of item means: 2, 6, 8, 9, 18, 29, 32, 37, 39 40, and 49)	31.3	44	Moving in the right direction	0.79
Student’s Academic Self-perceptions (sum of item means: 5, 10, 22, 26, 27, 31, 41 and 45)	20.2	32	Feeling more on the positive side	0.68
Students’ Perceptions of Atmosphere (sum of item means: 11, 12, 17, 23, 30, 33, 34, 35, 36, 42, 43 and 50)	29.9	48	A more positive attitude	0.81
Students’ Social Self-Perceptions (item means 3, 4, 14, 15, 19, 28 and 46)	17.1	28	Not too bad	0.72

**Table 3:** Confirmatory factor analysis of the DREEM by subscale

**Subscale**	**Item**	**Unstandardized factor loading**			**Standardized factor loading**		
		**Factor loading**	**Standard error**	**t-value**	**Factor loading**	**Standard error**	**t-value**
***Students’ perceptions of learning***							
	Q1	0.6239	0.1474	4.2320	0.3057	0.0636	4.8073
	Q7	1.4925	0.1784	8.3651	0.6789	0.0395	17.2052
	Q13	1.5760	0.1921	8.2029	0.6613	0.0410	16.1336
	Q16	1.1460	0.1380	8.3018	0.6720	0.0401	16.7727
	Q20	1.1417	0.1451	7.8664	0.6258	0.0440	14.2360
	Q21	1.1217	0.1550	7.2344	0.5627	0.0489	11.5139
	Q24	1.1284	0.1488	7.5834	0.5971	0.0463	12.9054
	Q25	0.9377	0.1863	5.0342	0.3692	0.0608	6.0761
	Q38	1.1364	0.1580	7.1945	0.5589	0.0492	11.3695
	Q44	1.5152	0.1902	7.9677	0.6364	0.0431	14.7670
	Q47	1.3846	0.2107	6.5728	0.5008	0.0532	9.4124
	Q48	1.3735	0.1782	7.7064	0.6095	0.0453	13.4582
***Students’ perceptions of faculty***							
	Q2	0.9471	0.1707	5.5478	0.4312	0.0588	7.3346
	Q6	1.2512	0.2332	5.3658	0.4144	0.0597	6.9396
	Q8	2.6053	0.3446	7.5599	0.6447	0.0440	14.6574
	Q9	2.5267	0.3653	6.9171	0.5698	0.0498	11.4454
	Q18	0.6911	0.2053	3.3669	0.2467	0.0670	3.6824
	Q29	2.5441	0.3336	7.6267	0.6529	0.0433	15.0756
	Q32	1.5668	0.2449	6.3969	0.5143	0.0537	9.5807
	Q37	1.8013	0.2452	7.3465	0.6190	0.0460	13.4430
	Q39	1.5016	0.2387	6.2913	0.5035	0.0544	9.2565
	Q40	1.2545	0.2119	5.9191	0.4665	0.0567	8.2243
	Q49	1.7067	0.2748	6.2098	0.4952	0.0549	9.0163
***Students’ academic self- perceptions***							
	Q5	0.6012	0.1221	4.9254	0.3497	0.0631	5.5419
	Q10	0.4707	0.0817	5.7588	0.4086	0.0602	6.7889
	Q22	1.1482	0.0966	11.8915	0.8251	0.0305	27.0709
	Q26	0.2701	0.0817	3.3063	0.2349	0.0675	3.4814
	Q27	0.8139	0.1087	7.4860	0.5303	0.0528	10.0414
	Q31	0.5603	0.0941	5.9548	0.4225	0.0594	7.1069
	Q41	0.6104	0.0866	7.0480	0.4995	0.0548	9.1071
	Q45	0.4088	0.0820	4.9855	0.3540	0.0629	5.6267
***Students’ perceptions of atmosphere***							
	Q11	0.6975	0.0894	7.7977	0.4994	0.0520	9.5969
	Q12	0.7920	0.1015	7.8063	0.4998	0.0520	9.6120
	Q17	0.3915	0.0923	4.2418	0.2845	0.0631	4.5067
	Q23	0.5747	0.0769	7.4694	0.4808	0.0532	9.0337
	Q30	0.5529	0.0753	7.3415	0.4735	0.0537	8.8208
	Q33	0.4993	0.0865	5.7701	0.3805	0.0589	6.4579
	Q34	0.5322	0.0759	7.0120	0.4546	0.0548	8.2879
	Q35	0.9709	0.0856	11.3359	0.6798	0.0382	17.8142
	Q36	0.7168	0.0930	7.7043	0.4941	0.0524	9.4342
	Q42	1.1167	0.1047	10.6660	0.6484	0.0409	15.8695
	Q43	1.0665	0.0901	11.8394	0.7025	0.0361	19.4404
	Q50	0.8264	0.1016	8.1352	0.5181	0.0508	10.2027
***Students’ social self- perceptions***							
	Q3	0.7794	0.0854	9.1271	0.6195	0.0464	13.3472
	Q4	0.9577	0.0880	10.8822	0.7233	0.0380	19.0504
	Q14	0.3078	0.0849	3.6231	0.2560	0.0669	3.8263
	Q15	0.5354	0.0832	6.4333	0.4473	0.0582	7.6908
	Q19	0.9263	0.0945	9.8048	0.6606	0.0432	15.3067
	Q28	0.8221	0.0941	8.7324	0.5951	0.0483	12.3271
	Q46	0.3790	0.0667	5.6826	0.3971	0.0609	6.5211

The overall score for the DREEM was 128.9 out of a possible 200 and the five subscale scores were; 1) students’ perceptions of learning 30.5 out of 48, 2) students’ perceptions of faculty 31.3 out of 44, 3) students’ academic self-perceptions 20.2 out of 32, 4) students’ perceptions of atmosphere 29.9 out of 48, and 5) students’ social self-perceptions 17.1 out of 28. The overall score and subscale scores, together with the interpretative descriptors recommended by the developers [26] are recorded in Table 2. Comparison of the overall score in the current study with those observed in other health science educational programs are summarized in Table 4.

**Table 4:** Comparison of the overall DREEM scores of health science educational programs

**Educational environment**	**Range of results for overall DREEM scores (number of studies)**	**Median score ± SD**	**References**
Medical education	89.9 – 153.3 (44 studies)	120.3 ± 15.85	[8,13,15,17,20,23,25,30-32],[34,37,39-64]
Dental education	111.6 – 123.1.0 (4 studies)	119.0 ± 5.78	[20,65-67]
Chiropractic	78.0 – 156.1 (4 studies)	105.5 ± 33.15	[14,16,24]
Nursing	104.0 – 132.5 (5 studies)	123.0 ± 10.41	[38,68,69]
Veterinary education (current study)	128.9 (1 study)	N/A	

The means and standard deviations for individual items of the DREEM are recorded in Table 5. Items were considered areas of concern if the mean item score was ≤ 2.0 [26]. Based on this threshold, four items were identified; “I am too tired to enjoy the program” (mean 1.79 ± 1.03), “the program is well scheduled” (mean 1.39 ± 0.99), “the teaching overemphasizes factual learning” (mean 1.67 ± 0.96), and “I am able to memorize all I need” (mean 1.31 ± 0.98).

**Table 5:** Mean and standard deviation for individual items of the DREEM

**Item**		**Mean**	**SD**
1	I am encouraged to participate during lectures	2.76	0.77
2	The lecturers are knowledgeable	3.52	0.52
3	There is a good support system for students who get stressed	2.27	0.98
***4***	***I am too tired to enjoy the program***^**†**^	***1.79***	***1.03***
5	Learning strategies which worked for me before continue to work for me now	2.19	1.10
6	The clinical faculty espouse a patient centered approach to clinical work	2.80	0.72
7	The teaching is often stimulating	2.49	0.83
8	The faculty ridicule the students^†^	2.90	0.96
9	The faculty are authoritarian^†^	2.25	1.06
10	I am confident about my passing this year	3.16	0.73
11	The atmosphere is relaxed during clinical teaching	2.20	0.87
***12***	***The program is well scheduled***	***1.39***	***0.99***
13	The teaching is student centered	2.51	0.90
14	I am rarely bored with the program	2.53	0.94
15	I have good friends within the program	3.31	0.93
16	The teaching helps to develop my competence	3.10	0.65
17	Cheating is a problem within the program^†^	3.10	0.86
18	The clinical faculty have good communication skills with clients	2.70	0.67
19	My social life is good	2.13	1.09
20	The teaching is well focused	2.73	0.69
21	I feel I am being well prepared for my profession	2.82	0.76
22	The teaching helps to develop my confidence	2.50	0.89
23	The atmosphere is relaxed during lectures	2.68	0.75
24	The teaching time is put to good use	2.70	0.72
***25***	***The teaching over emphasizes factual learning***^**†**^	***1.67***	***0.96***
26	Last year’s work has been a good preparation for this year’s work	2.67	0.73
***27***	***I am able to memorize all I need***	***1.31***	***0.98***
28	I seldom feel lonely	2.31	1.08
29	The faculty are good at providing feedback to students	2.46	0.93
30	There are opportunities for me to develop interpersonal skills	2.73	0.73
31	I have learned a lot about empathy in my profession	2.52	0.84
32	The faculty provide constructive criticism here	2.66	0.73
33	I feel comfortable in lectures socially	2.83	0.82
34	The atmosphere is relaxed in lectures or rounds	2.70	0.73
35	I find the experience disappointing^†^	2.95	0.89
36	I am able to concentrate well	2.37	0.91
37	The faculty give clear examples	2.82	0.69
38	I am clear about the learning objectives of the classes	2.81	0.77
39	The faculty get angry in lecture^†^	3.22	0.71
40	Faculty are well prepared for their teaching sessions	3.10	0.64
41	My problem solving skills are being well developed here	2.75	0.78
42	The enjoyment outweighs the stress of the program	2.21	1.08
43	The atmosphere motivates me as a learner	2.41	0.95
44	The teaching encourages me to be an active learner	2.44	0.90
45	Much of what I have to learn seems relevant to a career within veterinary medicine	3.03	0.73
46	My accommodation is pleasant	2.77	0.74
47	Long term learning is emphasized over short term learning	2.19	1.05
48	The teaching is too faculty centered^†^	2.38	0.86
49	I feel able to ask the questions I want	2.83	0.82
50	The students irritate the faculty^†^	2.27	1.00

SD, Standard deviation.

Items in bold and italics had a score ≤ 2.0.

^†^items that are negative statements with 4 = strongly disagree.

## Discussion

Over the last four decades there has been growing interest in students’ perceptions of their educational environment and the impact this may have on subsequent learning [19,25,29]. Educational research has demonstrated that the learning environment may influence student behaviour, academic achievement, course satisfaction and aspirations, as well as sense of well-being [6-11]. Therefore, evaluation of the educational environment is an important consideration for any programmatic or curricular review [12,14].

To our knowledge, this is the first time the DREEM has been applied within a veterinary educational setting and the first time within a United States healthcare professional program. Previously the DREEM has predominantly been used to evaluate the learning environments in undergraduate medical education programs in a variety of countries [19], but not the United States. Although medical and veterinary educational programs have many similarities, there are sufficient unique aspects of veterinary education to warrant investigation of the learning environment in this context. To this end, we wished to evaluate the internal reliability and construct validity of the overall DREEM tool and the five subscales, as well as identify items of concern as perceived by veterinary students.

In this study, the DREEM was shown to be a reliable tool in which to measure perceptions of the learning environment in a sample of veterinary students. Internal consistency was “excellent” [27] for the overall score of the DREEM with a Cronbach’s alpha = 0.93. This result is similar to the Cronbach’s alpha of the overall score in other studies which range from 0.87 - 0.93 [12,20,30-34]. The internal consistency of the subscales in this study were either “excellent” or “good” based on accepted guidelines [27], with the exception of students’ perception of academic-self which had an “acceptable” result of 0.67. The reasons for the lower internal consistency for academic self-perception can only be speculated, but may be due to the variable daily academic experiences of veterinary students affecting the consistency of their answers. Interestingly, other studies report more variable internal consistencies for the five subscales ranging from “good” (e.g., 0.80) to “poor” (e.g., 0.47), but no consistent pattern is observed [12,20,30-34]. This inconsistent reliability of the subscales in other studies has resulted in calls for analysis of the validity of the instrument [12].

The construct validity of the DREEM within this study was determined to be acceptable by confirmatory factor analysis, although the goodness of fit for the model was weak. Repetition of the study in our setting, as well as other veterinary programs, may allow better evaluation of the construct validity for the DREEM tool in veterinary medical education. Furthermore, repetition would allow better assessment of individual items in the tool, including whether they should be omitted or re-written, potentially strengthening construct validity. Other studies have evaluated the psychometric properties of the DREEM and found variable results for construct validity, as well as subscale reliability [12,20,30-34]. This variation between studies emphasizes the need for continued psychometric evaluation of the DREEM to better determine its suitability for evaluation of students’ perceptions of their educational environment, as well as comparison of these environments in a variety of health education settings.

After the internal consistency and construct validity of the DREEM was determined in this study, descriptive interpretations of quantitative results were applied as outlined by the developers of the tool [26]. Using these recommendations, the overall score of 128.9 observed in the current study can be interpreted as “more positive than negative”. While this is below the highest designation for this category (Table 1), our overall score for students’ perceptions was above the median overall score for studies conducted in medical, dental, nursing and chiropractic programs (Table 4). As with the overall score, the interpretation of subscale scores for veterinary student perceptions’ were positive in all five subscales based on the developers’ descriptors (Table 2). Comparison of these results to other studies is more difficult as there are no consistent findings for specific subscales between studies, with positive and negative results recorded for all subscales across a variety of health sciences programs.

A threshold mean score of ≤2.0 is used to interpret individual items based on recommendations of the DREEM developers. Using this threshold, four items of concern were identified in this study. The four items were; (1) “I am unable to memorize all I need” (mean = 1.31 ± 0.98), (2) “the program is well scheduled” (mean = 1.39 ± 0.99), (3) “the teaching over emphasises factual learning” (mean = 1.67 ± 0.96), and (4) “I am too tired to enjoy the program” (mean = 1.79 ± 1.03). It is important to note that these four items are derived from different subscales, but all relate to a common theme of workload. Furthermore, this finding supports earlier studies describing concerns about expanding curricula in veterinary medical education and the impact on student learning [5,35,36]. These results are worrying, as factors impeding the academic progress of veterinary students may include lack of motivation, stress, and fatigue [36]. These factors all can be related to a curriculum’s heavy workload and intensity [35]. A further consequence of this perceived heavy workload may be “reproduction of information rather than construction of knowledge” [5]. Interestingly, students in medical and dental programs have similar concerns regarding their ability to “memorize all they need” as reported in other DREEM studies [20,37]. It therefore appears that a consistent theme within healthcare education is that workload is perceived as being excessive and this perception may have a negative impact on the learning environment.

Solutions to decrease curricula load in veterinary medical education could involve deleting redundant content by focusing on Day-1 competencies; consideration of limited licensure (i.e., veterinarians licensed to practice on fewer species such as dogs/cats only) allowing for decreased curricula content; or lengthening the time of training to include an internship year during which students are not licensed to practice. Each solution has advantages and disadvantages but all would lead to widespread curricula reform and would require agreement amongst major stakeholders including the American Veterinary Medical Association as well as veterinary testing, licensing, and accreditation bodies within the United States.

A further consideration regarding students’ negative perceptions of scheduling is that these concerns are likely multifactorial and could relate to contact hours, timing of assessments, errors in timetables, or equity of schedules for different groups. In our situation, schedules have been created manually which has allowed for multiple errors in the schedule. Based on students’ feedback, this appeared to be a major source of concern and frustration. Although this problem has been addressed with the introduction of an electronic scheduling program, it highlights the negative impact administrative processes may have on the learning environment.

One potential issue with the current study is the relatively low response rate (53%) achieved. Variable response rates have been reported in other DREEM studies ranging from 48% to 97% [38,39]. However, many studies do not report a response rate, rather the number of students responding to the survey, making comparison of response rates difficult. The response rate in the current study was impacted by the low participation of students in the final year where these students are undergoing their clinical training and are therefore distributed around the United States. Furthermore, the lower response rate of the students in the clinical year may have negatively impacted the DREEM scores, as these students frequently have a more positive outlook on their educational environment due to their immersion in workplace-based learning. To address this issue we intend to repeat this survey in the coming year and will administer the survey immediately before graduation when all final year students have returned to the college. In addition, the DREEM survey will be administered to students in the pre-clinical years as part of a course which should improve their response rate.

## Conclusions

This study has demonstrated that the DREEM can be applied in a veterinary medical context to provide reliable and valid information on veterinary students’ perceptions of their learning environment. Furthermore, the study identified student concerns regarding workload. As veterinary educators, it is interesting to note that other health education programs encounter similar challenges and concerns in their learning environments. This highlights the utility in sharing information and solutions regardless of the specific program. Similarly, we believe it would be beneficial for other veterinary programs worldwide to apply the DREEM to allow comparison between programs in this field. Sharing this information could lead to better development of best practices in veterinary student learning environments.

## Competing interest

The authors declare that they have no competing interests.

## Authors’ contributions

JMP conceptualized the idea for this study and administered the tool to the veterinary students. JMP and JLH contributed to the drafting and final development of this manuscript. SRW conducted the statistical analyses and provided input into the relevant sections of the manuscript. All authors read and approved the final manuscript.

## Authors’ information

JMP is the Director of Admissions and Student Services at the Virginia Maryland Regional College of Veterinary Medicine (VMRCVM). Her interests include teaching and assessing professional competencies and admissions policies and procedures. JLH is the Associate Dean for Professional Programs at the VMRCVM. Her interests include curriculum design and program development. SRW is a Research Assistant Professor at the VMRCVM. His interests include psychometric analysis of research data.
